# Ancestral risk modification for multiple sclerosis susceptibility detected across the Major Histocompatibility Complex in a multi-ethnic population

**DOI:** 10.1371/journal.pone.0279132

**Published:** 2022-12-22

**Authors:** Ashley H. Beecham, Lilyana Amezcua, Angel Chinea, Clara P. Manrique, Lissette Gomez, Andrea Martinez, Gary W. Beecham, Nikolaos A. Patsopoulos, Tanuja Chitnis, Howard L. Weiner, Philip L. De Jager, Esteban G. Burchard, Brett T. Lund, Kathryn C. Fitzgerald, Peter A. Calabresi, Silvia R. Delgado, Jorge R. Oksenberg, Jacob L. McCauley

**Affiliations:** 1 John P. Hussman Institute for Human Genomics, Miller School of Medicine, University of Miami, Miami, FL, United States of America; 2 Dr. John T. Macdonald Department of Human Genetics, Miller School of Medicine, University of Miami, Miami, FL, United States of America; 3 Department of Neurology, Keck School of Medicine, University of Southern California, Los Angeles, CA, United States of America; 4 San Juan MS Center, Guaynabo, Puerto Rico, United States of America; 5 Ann Romney Center for Neurological Diseases, Brigham and Women’s Hospital, Boston, MA, United States of America; 6 Center For Translational & Computational Neuroimmunology and the Multiple Sclerosis Center, Department of Neurology, Columbia University Irving Medical Center, New York, NY, United States of America; 7 Department of Bioengineering and Therapeutic Sciences, University of California, San Francisco, San Francisco, CA, United States of America; 8 Department of Neurology and The Solomon H. Snyder Department of Neuroscience, Johns Hopkins University School of Medicine, Baltimore, MD, United States of America; 9 Multiple Sclerosis Division, Department of Neurology, Miller School of Medicine, University of Miami, Miami, FL, United States of America; 10 Department of Neurology, University of California, San Francisco, San Francisco, CA, United States of America; Oklahoma Medical Research Foundation, UNITED STATES

## Abstract

The Major Histocompatibility Complex (MHC) makes the largest genetic contribution to multiple sclerosis (MS) susceptibility, with 32 independent effects across the region explaining 20% of the heritability in European populations. Variation is high across populations with allele frequency differences and population-specific risk alleles identified. We sought to identify MHC-specific MS susceptibility variants and assess the effect of ancestral risk modification within 2652 Latinx and Hispanic individuals as well as 2435 Black and African American individuals. We have identified several novel susceptibility alleles which are rare in European populations including HLA-B*53:*01*, and we have utilized the differing linkage disequilibrium patterns inherent to these populations to identify an independent role for HLA-DRB1*15:*01* and HLA-DQB1*06:*02* on MS risk. We found a decrease in Native American ancestry in MS cases vs controls across the MHC, peaking near the previously identified *MICB* locus with a decrease of ~5.5% in Hispanics and ~0.4% in African Americans. We have identified several susceptibility variants, including within the *MICB* gene region, which show global ancestry risk modification and indicate ancestral differences which may be due in part to correlated environmental factors. We have also identified several susceptibility variants for which MS risk is modified by local ancestry and indicate true ancestral genetic differences; including HLA-DQB1*06:*02* for which MS risk for European allele carriers is almost two times the risk for African allele carriers. These results validate the importance of investigating MS susceptibility at an ancestral level and offer insight into the epidemiology of MS phenotypic diversity.

## Introduction

The Major Histocompatibility Complex (MHC), located on chromosome 6p21.3, is vital to proper immune system function due to its central role in the initiation of adaptive immune response. Faulty reactions may result in destruction of normal tissue and manifest as an autoimmune disease such as multiple sclerosis (MS); a neurodegenerative disease characterized by transmigration of peripheral autoreactive leukocytes into the central nervous system. The MHC makes the single largest genetic contribution to MS susceptibility in whites of European ancestry, on its own explaining ~20% of the heritability estimated from genotyped SNPs [1] and was first identified as a determinant of MS risk in the 1970s by utilizing lymphocyte cultures [2] and lymphocytotoxic antisera reactions [3]. The most extensively studied and replicated association has been seen with HLA-DRB1*15:*01*, demonstrating the strongest genetic effect in European [1] and African American individuals [4]. Early genome-wide association studies (GWAS) in populations of European ancestry confirmed the effect of HLA-DRB1*15:*01* (risk, class II) and identified HLA-A*02:*01* (protective, class I), HLA-DRB1*03:*01* (risk, class II), and HLA-DRB1*13:*03* (risk, class II) as susceptibility loci within the MHC [5]. Subsequent analyses extended this list further only in populations of European ancestry [1, 6, 7]. Presently 32 statistically independent additive and dominant effects across class I, class II, and non-HLA genes within the MHC complex have been identified [1]. Prior evidence has also pointed to the effect of interactions on MS risk; both epistatic interactions between MHC alleles [8] and environmental interactions with MHC alleles [9], highlighting the complexity of the role of the MHC on disease susceptibility.

The MHC is both polygenic (containing a variety of genes with a range of binding specificity) and highly polymorphic (containing multiple variants within each gene), making antigen evasion difficult. While being polygenic allows an individual to respond to a wide array of antigens (i.e. ensuring survival of an individual), polymorphism ensures the capture of antigens at a species-wide level (i.e. ensuring survival of a species) [10]. While this variability is crucial from a biological standpoint, the long-range linkage disequilibrium (LD) and extensive allelic heterogeneity inherent to the region have made refining the MS associated risk signal to the underlying causal variants a difficult endeavor. MHC variation across ancestral populations is also high, with numerous population-specific alleles and allele frequency differences noted [10]. Therefore, the 32 susceptibility alleles identified in populations of predominantly European ancestry may not represent the most relevant susceptibility alleles across the MHC in all ancestrally distinct populations. In fact, a study in 2004 demonstrated that the HLA-DRB*15:*03* allele, which occurs almost exclusively in individuals of African descent, confers moderate risk to MS in African American individuals but no measurable risk in other populations [11, 12]. Previous studies in ancestrally diverse Latin American populations, across various countries of origin, were small in size and demonstrated inconclusive results [13–18]. While prevalence of MS has traditionally been considered lower in Latinx and Hispanic individuals and Black and African American individuals than in individuals of European ancestry; epidemiological evidence now indicates that prevalence may be more similar between populations than previously indicated, and clinical manifestations are diverse [19]. A detailed investigation of this region in ancestrally admixed individuals formed through interbreeding of populations, including Latinx and Hispanic individuals as well as Black and African American individuals, could serve to both uncover the MHC risk in these underrepresented populations and more definitely chronicle the ancestral lineage of MHC haplotypes.

Our objective is therefore to test for the association of genetic variation across the extended MHC (chromosome 6 from 29–34 MB) with MS in a multi-ethnic cohort of self-identified Latinx and Hispanic individuals (collectively we refer to them as Hispanic) and Black and African American individuals (collectively we refer to them as African American) and then to assess the effect of ancestral allele origin on risk.

## Materials and methods

### Study population

2995 self-reported Hispanic individuals (1558 with MS, 1437 controls) and 2630 self-reported African American individuals (1427 with MS, 1203 controls) were ascertained from seven US participating institutions as part of the Alliance for Research in Hispanic MS (ARHMS) Consortium. Following all quality control, a total of 2652 unrelated Hispanics (1298 MS cases and 1354 controls) and 2435 unrelated African Americans (1298 MS cases and 1137 controls) remained for analysis. A detailed review of the quality control process and a breakdown of samples by ascertainment site have been previously described [20]. The institutional review boards at each institution approved this study, and all participants provided written informed consent prior to participation.

We additionally obtained DNA on 498 European (EUR), 379 African (AFR), 180 East Asian (EAS), and 299 Central and South-American Hispanic (AMR) samples from the 1000 Genomes Project [21] to be used for quality control. A total of 436 EUR, 318 AFR, 160 EAS, and 262 AMR remained after exclusion of individuals with low call rate (≤98%), excess autosomal heterozygosity ≥3 SD from the mean, and excess identity by descent signifying a sample duplication or relatedness (proportion IBD > 0.2).

### Genotype calling and SNP quality control

All DNA were obtained through whole blood extraction. DNA were genotyped on the MS Chip, an Illumina Infinium custom genotyping array which contains targeted and dense coverage of the extended MHC, specifically designed for imputation [1, 20]. All sample genotyping was conducted by the Center for Genome Technology within the John P. Hussman Institute for Human Genomics at the University of Miami. Genotype calling was done using GenomeStudio 2.0, and manual review was done for all 17,963 variants across the extended MHC.

Variants were excluded within Hispanics, African Americans, and each 1000 Genome population separately based on poor performing clusters, low call rate (CR) with respect to minor allele frequency (MAF): CR ≤ 99.5% when MAF ≤ 5%, CR ≤ 99% when 5% ≤ MAF ≤ 10%, and CR ≤ 98% when MAF > 10%, and discordance between plate controls (one genotyping control on each 96-well plate). Within the Hispanic and African American study samples, variants were additionally excluded if they were out of Hardy-Weinberg equilibrium (chi-square p ≤ 1.00 x 10^−05^ in disease controls) or were differentially missing between those with MS and controls (p ≤ 1.00 x 10^−03^). In total, a common set of 9909 SNPs remained across the MHC in the Hispanic and African American study sample following all quality control; 8856 in EUR; 8859 in AFR; 8848 in EAS, and 8877 in AMR.

### Imputation and accuracy assessment of classical HLA alleles

Classical HLA alleles, SNPs, and amino acid residues were imputed simultaneously from genotyped SNPs using HLA-TAPAS and a multi-ethnic reference panel of 2504 samples from the 1000 Genome Project (503 EUR, 661 AFR, 347 AMR, 504 EAS, 489 SAS) for each of the Hispanic and African American samples and for a subset of the genotyped 1000 Genomes Project samples which were non-overlapping with the multi-ethnic reference panel (100 AFR, 100 AMR, 100 EUR, 153 EAS) [22]. Additionally, using the same multi-ethnic reference panel, HLA-TAPAS imputation was performed on Illumina Multi-Ethnic Genotyping Array (MEGA) data for 40 unrelated Native American (NAM) samples from the Human Genome Diversity Project (HGDP) which was provided to us through the PAGE Consortium [23]. These data included individuals from the Surui and Karitiana populations of Brazil, Maya population of Mexico, and a native population of Columbia. At the variant level; alleles, SNPs, and amino acid residues imputed with allelic R-squared less than 0.4 were removed from further analysis. In addition, individual genotypes with an estimated genotype probability (GP) of less than 0.8 were zeroed, and subsequently variants with call rate less than 95% were removed.

To assess the validity of HLA-TAPAS imputation, publicly available classical HLA types produced by colleagues at the University of California, San Francisco [24] and hosted by the International Genome Sample Resource were downloaded for the subset of 1000 Genomes Project samples which we also imputed. HLA typing methodology for the 1000 Genomes samples has been described in detail previously [24]. Concordance between the HLA types produced from classical Sanger sequencing and the HLA types imputed from HLA-TAPAS for individuals from AFR, AMR, EUR, and EAS was assessed to determine imputation reliability in diverse populations. In addition, HLA types from Sanger sequencing were available for 615 of our African American cases, and imputation accuracy was assessed in the same way (typing methodology indicated in S1 Table). Specifically, within the African American and 1000 Genomes Project samples with Sanger validated allele calls; rate of quality imputation was first assessed as the number of alleles which were successfully imputed and passed quality control (i.e. quality alleles) divided by the number of possible imputed alleles. Concordance rates were then assessed as the number of quality alleles that were concordant with the Sanger allele calls divided by the total number of quality alleles.

### Ancestry estimation

To evaluate local ancestry, a phased set of 20,048 genotyped or imputed MHC variants which were non-monomorphic and passed all quality control in Hispanic, African American, and HGDP Native American samples were generated with HLA-TAPAS. Local ancestry was then assessed in Hispanic and African American samples using RFMIXV2 [25] with phased reference panels of Native Americans (40 HGDP), Europeans (40 CEU 1000G), and Africans (40 YRI 1000G). Equal sized reference panels were chosen to ensure no bias was introduced into the estimation of local ancestry. Local ancestry was evaluated as the number of African (AFR), European (EUR), and Native American (NAM) haplotypes seen at each variant position (0, 1, or 2 for an individual). Global ancestry was evaluated as the proportion of ancestry from each reference population with ADMIXTURE [26] using the same reference panel and a set of genome-wide tagging SNPs consisting of 10,928 independent non-MHC SNPs (R^2^ ≤ 0.2) which were not within 1-Mb of any previously identified MS risk variant [1], and passed quality control in the Hispanic, African American, and HGDP NAM samples.

### Analysis of classical HLA alleles and SNPs

Marginal association between MS status and each of the classical HLA alleles, SNPs, and amino acid residues (collectively termed as variation within the MHC) was assessed using logistic regression in the Hispanic and African American study sample, adjusting for global ancestry proportions to account for population substructure.

Forward stepwise conditional logistic regression was used to identify statistically independent effects. The primary classical allele was included as a covariate, and the association analysis was repeated for the remaining classical alleles; allowing for additive, dominant, and recessive effects at each step. This process was performed again until no classical alleles reached the minimum suggestive level of significance (p-value <1 × 10^−03^). Following the inclusion of all qualified classical alleles, the process was continued to allow for inclusion of SNPs or amino acid residues to a more stringent threshold of p-value <1 × 10^−04^; again allowing for additive, dominant, and recessive effects.

### Assessing the effect of sex on MS risk

To determine whether sex modifies the effect of variation on MS risk, we used a logistic regression model to evaluate the effect of an interaction between sex and each independent risk variant using the likelihood ratio test (LRT). A full model (Eq 1) was compared to a reduced model without the interaction term; where *G*_*EUR*_ and *G*_*NAM*_ represented the global European and Native American ancestry components and *Allele* represented the number of copies (0, 1, or 2) of each independent risk allele.

$$logit(MS)=\beta_{0}+\beta_{1}Allele+\beta_{2}G_{EUR}+\beta_{3}G_{NAM}+\beta_{4}Sex+\beta_{5}Allele*Sex$$ (1)

When a sex interaction was observed (p < 0.05), sex stratified analyses were performed.

### Assessing the effect of ancestry on MS risk

To determine the contribution of local ancestry to MS risk across the MHC, we used the following model (Eq 2); where *L*_*EUR*_ and *L*_*NAM*_ represent the local ancestry components. The association of local ancestry with MS risk was assessed using a LRT to compare the full model to a restricted model which excluded local ancestry.


$$logit(MS)=\beta_{0}+\beta_{1}G_{EUR}+\beta_{2}G_{NAM}+\beta_{3}L_{EUR}+\beta_{4}L_{NAM}$$ (2)

To determine whether ancestry modifies the effect of genetic variation on MS risk, we used two logistic regression-based models. The first model evaluated the role of global ancestry on risk variation by evaluating the effect of an interaction between global ancestry and each independent risk variant using the LRT. A full model (Eq 3) was compared to a reduced model without the interaction terms.

$$logit(MS)=\beta_{0}+\beta_{1}Allele+\beta_{2}G_{EUR}+\beta_{3}G_{NAM}+\beta_{4}Allele*G_{EUR}+\beta_{5}Allele*G_{NAM}$$ (3)

When a global ancestry interaction was observed (p < 0.05), a graphical model was used to illustrate the variant-specific ancestral differences in MS risk by first computing the estimated odds of risk using varying scenarios of global ancestry proportion and copies of the MS associated allele. Specifically, within the Hispanic sample the estimated odds of MS (Eq 3) were computed by holding the proportion of AFR ancestry constant at the average of 11% observed in our sample and varying the NAM ancestry proportion using the maximum, mean, and minimum observed in our sample (82%, 15%, and 0% respectively). In this way, estimated odds of risk were computed for each of 0, 1, and 2 copies of the variant allele under three different ancestral scenarios and plotted linearly to illustrate changes in effect. Similarly, within the African American sample, the estimated odds of MS were computed by holding the proportion of NAM constant at the average of 2% observed in our sample and varying the AFR ancestry proportion using the observed maximum, mean, and minimum (98%, 78%, and 16% respectively).

The second model evaluated the role of local ancestry on risk variation by evaluating the effect of an interaction between local ancestry and each independent risk variant. Again, a full model (Eq 4) was compared to a reduced model without the interaction terms; where *L*_*STATE*_ represented the local ancestry state at the corresponding variant position (EUR∼EUR,EUR∼AFR,EUR∼NA,AFR∼AFR,AFR∼NA,NA∼NA as the six possible ancestral states for each phased allele).

$$logit(MS)=\beta_{0}+\beta_{1}Allele+\beta_{2}G_{EUR}+\beta_{3}G_{NA}+\beta_{4}L_{STATE}+\beta_{5}Allele*L_{STATE}$$ (4)

When a local ancestry interaction was observed (p < 0.05), a haplotype model was used to assess MS risk in an allele-specific manner. By testing for presence of an allele from a certain ancestral background against absence of the allele on the same ancestral background, we were able to estimate effect sizes for ancestry-specific haplotypes. Specifically, each allele was first grouped by local ancestry state and then within each ancestral group, the effect of the allele on MS risk was assessed using generalized estimating equations (GEE). Individual was used as the grouping variant, and adjustment for global ancestry was made. A global ancestry interaction in the absence of a local ancestry interaction would be taken as evidence that the ancestral interaction may be due to highly correlated environmental factors rather than to genetic ancestry alone.

## Results

### Distribution of ancestry

Following all quality control, a total of 2652 Hispanics (1298 MS cases and 1354 controls) and 2435 African Americans (1298 MS cases and 1137 controls) remained for analysis. As previously described, Hispanic MS cases and controls in this dataset are on average 74% European, 15% Native American, and 11% African [20]. There is slightly less European ancestry and slightly more Native American ancestry in individuals with MS compared to controls (71% vs 76% respectively for European, p = 3.59 x 10^−12^; 18% vs 13% respectively for Native American, p = 3.61 x 10^−16^). Geographical differences in ancestry are observed; with a greater proportion of Native American ancestry identified in Hispanics residing on the west coast (39% vs 11%) and a greater proportion of European ancestry in Hispanics residing on the east coast of the United States (79% vs 54%); in accordance with relocations of native populations that occurred with European colonization of the Americas [27]. The African Americans on the other hand, have a non-zero proportion of Native American ancestry and are on average 78% African, 20% European, and 2% Native American, with similar proportions in cases and controls and across geographical regions, as previously described [20].

### Imputation accuracy of HLA alleles

While imputation accuracy varied by allele and by population, concordance rates remained high following our stringent quality filters. On average across populations, quality rates were lowest for *HLA-DRB1* (59%) and highest for *HLA-C* (94%), and concordance rates were lowest for HLA-DRB1 (93%) and highest for HLA-DQB1 (99%) (S1 Table). For 1000 Genomes Africans (AFR), concordance rates ranged from 92% for *HLA-DRB1* to 98% for *HLA-B*, with an average of 95% across alleles. For 1000 Genomes Project Hispanics (AMR), concordance rates ranged from 95% for *HLA-DRB1* to 99% for *HLA-DQB1*, with an average of 98% across alleles. Most notably, concordance rates also exceeded 90% across all alleles in our African American case sample, ranging from 90% for *HLA-DRB1* to ~100% for *HLA-DQB1* (S1 Table).

### Associations with MS risk

**Fig 1:**
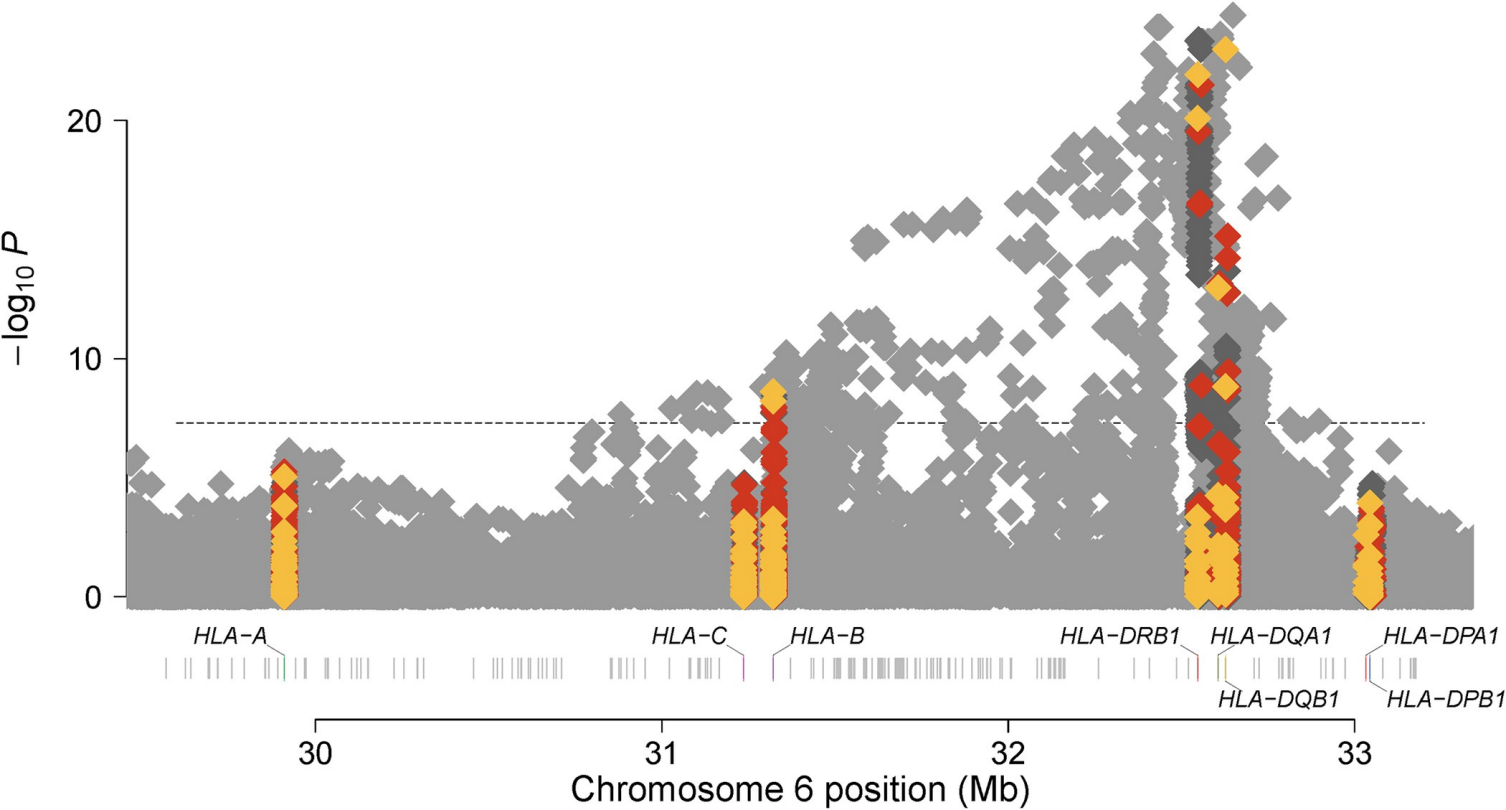
Manhattan plot for marginal additive association between variation within the MHC and MS risk in the Hispanic sample. Genomic position (HG19) is denoted on the x-axis and -log(10) P-value for the association of an additive effect of each SNP (grey), classical allele (yellow), and amino acid (orange) with MS risk on the y-axis.

**Fig 2:**
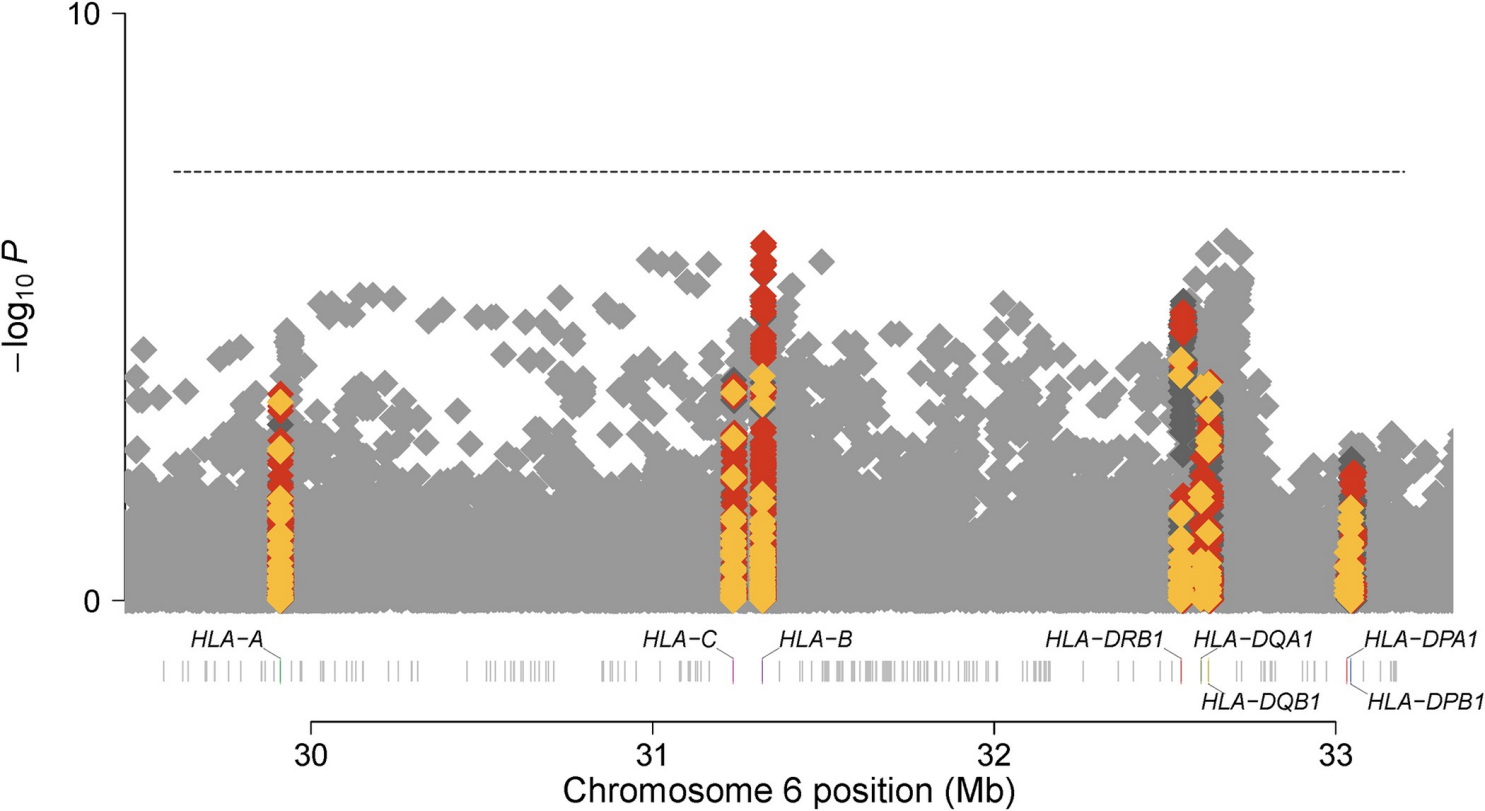
Manhattan plot for the marginal additive association between variation within the MHC and MS risk in the African American sample. Genomic position (HG19) is denoted on the x-axis and -log(10) P-value for the association of an additive effect of each SNP (grey), classical allele (yellow), and amino acid (orange) with MS risk on the y-axis.

In the marginal analysis of classical alleles (Figs 1 and 2), HLA-DQB1*06:*02* indicated the strongest additive association with MS risk in Hispanics (additive OR = 2.52, p = 1.05 x 10^−23^) (S2 Table); while HLA-DRB1*15:*01* indicated the strongest additive association with MS risk in African Americans (additive OR = 1.81, p = 8.00 x 10^−05^) (S3 Table). While linkage disequilibrium is extremely high between HLA-DQB1*06:*02* and HLA-DRB1*15:*01* in European populations (R2 > 0.9 in 1000 Genomes Project Europeans); it is lower in Hispanic (R2 = 0.65) and African American controls (R2 = 0.15) and thus provides an ideal setting to determine the risk attributable to each allele.

Stepwise conditional modeling indicated that after conditioning on the stronger dominant effect of HLA-DQB1*06:*02* in Hispanics (dominant OR = 2.70, p = 2.66 x 10^−24^), a residual association of HLA-DRB1*15:*01* with MS risk remained (additive OR = 1.83, p = 9.63 x 10^−04^*)* (Table 1). Similarly, after conditioning on the stronger dominant effect of HLA-DRB1*15:*01* in African Americans (dominant OR = 1.91, p = 3.48 x 10^−05^); although not meeting the stringent threshold of p < 1.00 x 10^−03^ for secondary signals, a residual association of HLA-DQB1*06:*02* with MS risk remained (dominant OR = 1.25, p = 1.57 x 10^−02^).

**Table 1:** Independent classical HLA alleles and SNPs in Hispanics.

Allele/SNP	Model	Freq (Allele)	OR (L95-U95)‡	P	Amino Acid in LD (R2>0.8)	Best SNP Tag	SNP R2	Gene	Function
DQB1*06:*02*	Dom	0.08	2.70 (2.23–3.27)	2.66E-24	None	rs9273342	0.99		
A*02:*01*	Add	0.19	0.69 (0.59–0.80)	2.72E-06	A_95_29911057_exon3_V	rs2844821	0.89		
DPB1*03:*01*	Add	0.07	1.53 (1.24–1.87)	5.36E-05	None	DPB1-2939-33046757-intron1	0.85		
DRB1*13:*03*	Add	0.02	2.25 (1.52–3.34)	5.05E-05	None	rs75589097	0.94		
DQB1*02:*01*	Add	0.21	1.36 (1.18–1.57)	2.80E-05	(DQB1)* _-10_32634317_exon1_S; _74_32632630_exon2_A; _71_32632639_exon2_K; _55_32632687_exon2_L; _37_32632741_exon2_I; _30_32632762_exon2_S; _-10_32634317_exon1_A; _37_32632741_exon2_Y	DQB1-6203-32628182-intron5	1		
DRB1*15:*01*	Add	0.07	1.83 (1.28–2.62)	9.63E-04	None	rs9269243	0.99		
rs371143509	Dom	0.09 (T)	0.55 (0.43–0.71)	2.44E-06	None			*MICB*	intron
rs6929950†	Add	0.04 (C)	0.42 (0.29–0.61)	6.06E-06	None			*OR5V1*	intron
rs112394499	Dom	0.10 (T)	1.62 (1.32–2.01)	6.82E-06	None			*HCG24*	nc-transcript
rs2844503	Add	0.48 (A)	1.34 (1.17–1.52)	1.72E-05	None			*HCG26 / MICB-DT*	intergenic
rs6902067	Rec	0.34 (A)	0.52 (0.39–0.71)	3.46E-05	None			*MICA / MICB*	intergenic
rs3021302	Rec	0.14 (C)	3.18 (1.83–5.52)	4.16E-05	None			*HLA-DQA1 / DQB1*	intergenic

† indicates a novel variant in Hispanics

Model: Add = Additive, Dom = Dominant, Rec = Recessive

‡ Reported odds ratios and 95% confidence intervals are sequential as identified in the stepwise regression model such that the effect size for each allele/SNP is reported after conditioning on the alleles/SNPs in the preceding rows

Gene has been annotated with ANNOVAR version date June 2020 (Wang et al., *Nucleic Acids Research*, 2010)

In total, stepwise conditional modeling indicated 12 independent signals (p ≤ 1.00 x 10^−03^) across the MHC in Hispanics; 6 classical alleles (HLA-DQB1*06:*02* dominant, risk; HLA-A*2:*01* additive, protective; HLA-DPB1*03:*01* additive, risk; HLA-DRB1*13:*03*:*01* additive, risk; HLA-DQB1*02:*01* additive, risk; HLA-DRB1*15:*01* additive, risk) and 6 SNPs (Table 1). All of the classical alleles have been previously identified in Europeans [1]. Four of the six SNPs are in minimal LD with previously identified effects [1] (R2 ranging between 0.1 and 0.2 in both Hispanic controls and European populations); including rs371143509, rs2844503, and rs6902067 which are all within or flanking *MHC class I polypeptide-related sequence B* (*MICB*) as well as rs112394499, a non-coding transcript variant within *HLA complex group 24* (*HCG24*). The fifth SNP (rs3021302) is in minimal LD (R2 = 0.15 in Hispanic controls) with the previously identified classical allele, HLA-DQB1*02:*01*; however, residual signal remains for rs3021302 after conditioning (p = 4.16 x 10^−05^). Lastly, rs6929950, an intronic variant within olfactory receptor family 5 subfamily V member 1 (*OR5V1*) represents a novel association with MS risk in Hispanics and has a frequency ranging from <0.01 in gnomAD Europeans to 0.10 in gnomAD Africans (S4 Table).

In African Americans, stepwise conditional modeling indicated 6 independent signals; 3 classical alleles (HLA-DRB1*15:*01* dominant, risk; HLA-A*02:*01* additive, protective; HLA-B*53:*01* dominant, protective) and 3 SNPs (Table 2). While HLA-DRB1*15:01 and HLA-A*2:*01* have previously been identified for association with MS; HLA-B*53:*01* represents a novel association in African Americans and has a frequency less than 1% in Europeans (S4 Table). Two of the three SNPs have minimal to moderate LD with previously identified effects; rs2516423 which flanks *MICB* (R2 = 0.20 with previously identified rs2523500 [1] in African American controls and R2 < 0.1 in Europeans) and rs28371315, an intergenic variant which tags the previously identified amino acid position 221 within exon 4 of *DQB1* [1] (R2 = 0.44 in African American controls and R2 = 0.27 in Europeans). Lastly, rs760145, an intronic SNP within *HLA-F antisense RNA 1 (HLA-F-AS1)* represents a novel association in African Americans.

**Table 2:** Independent classical HLA alleles and SNPs in African Americans.

Allele/SNP	Model	Freq (Allele)	OR (L95-U95)‡	P	Amino Acid in LD (R2>0.8)	Best SNP Tag	SNP R2	Gene	Function
DRB1*15:*01*	Dom	0.03	1.91 (1.41–2.59)	3.48E-05	none	rs9269243	0.99		
A*02:*01*	Add	0.13	0.68 (0.57–0.82)	5.07E-05	AA_95_29911057_exon3_V	rs12153924	0.98		
B*53:*01*	Dom	0.12	0.68 (0.55–0.84)	3.22E-04	none	rs115219755	0.92		
rs760145	Add	0.45 (T)	0.75 (0.66–0.85)	7.75E-06	none			*HLA-F-AS1*	Intron
rs28371315	Rec	0.34 (C)	0.52 (0.39–0.70)	8.85E-06	none			*HLA-DQB1 / DQA2*	Intergenic
rs2516423	Dom	0.50 (A)	0.64 (0.53–0.79)	1.59E-05	none			*MICB-DT*	intron

‡ indicates a novel variant in African Americans

Model: Add = Additive, Dom = Dominant, Rec = Recessive

‡ Reported odds ratios and 95% confidence intervals are sequential as identified in the stepwise regression model such that the effect size for each allele/SNP is reported after conditioning on the alleles/SNPs in the preceding rows

Gene has been annotated with ANNOVAR version date June 2020 (Wang et al., *Nucleic Acids Research*, 2010)

### Sex modification of MS risk

One of the twelve independent MHC variants identified for association with MS in the Hispanic sample demonstrated risk modification by sex (Table 3). In a marginal analysis, females with HLA-DQB1*02:*01* were at significant risk for MS (OR = 1.24, p = 1.72 x 10^−02^), while no significant effect was observed for males (Table 4). Two of the six independent MHC variants identified for association with MS in the African American sample demonstrated risk modification by sex; HLA-A*02:*01* and rs2516423 (Table 3). A highly significant protective effect for HLA-A*02:*01* was observed in females (OR = 0.63, p = 1.21 x 10^−05^), while no effect was seen in males (Table 4). Conversely, a highly protective effect for rs2516423 was observed in males (OR = 0.55, p = 1.82 x 10^−03^), while no effect was seen in females (Table 4). We found no significant difference in risk for females and males with HLA-DRB1*15:*01* (interaction p > 0.05 in both Hispanics and African Americans, Table 3); whereas it has been suggested in other studies that HLA-DRB1*1501 is more prevalent in females [28, 29] and that females may confer a higher HLA-DRB1*15:*01* specific risk [30].

**Table 3:** Interactions with MHC risk variation.

			Global Interaction		Local Interaction		Sex Interaction	
Alleles/SNPs	Pos Hg19 (MB)	Population	HISP P	AA P	HISP P	AA P	HISP P	AA P
A*02:*01*		HISP + AA	2.23E-01	7.92E-01	1.40E-01	**4.14E-02**	8.15E-01	**2.33E-02**
B*53:*01‡*		AA	4.14E-01	6.68E-01	**4.37E-02**	7.02E-02	6.51E-01	3.80E-01
DRB1*13:*03*		HISP	1.82E-01	1.84E-01	3.04E-01	5.35E-01	3.60E-01	6.87E-01
DRB1*15:*01*		HISP + AA	9.68E-01	3.42E-01	3.47E-01	**5.09E-02**	3.44E-01	1.53E-01
DQB1*02:*01*		HISP	6.17E-01	4.49E-01	8.62E-01	2.89E-01	**1.85E-02**	1.99E-01
DQB1*06:*02*		HISP	1.71E-01	1.02E-01	**4.33E-02**	**9.62E-03**	8.35E-01	1.62E-01
DPB1*03:*01*		HISP	6.62E-01	2.65E-01	2.08E-01	2.00E-01	8.23E-01	9.60E-01
rs6929950†	29.4	HISP	6.44E-01	4.87E-01	2.01E-01	**2.72E-02**	1.98E-01	2.76E-01
rs760145‡	29.7	AA	2.73E-01	**1.45E-03**	2.13E-01	2.60E-01	8.49E-01	4.79E-01
rs6902067	31.4	HISP	6.43E-01	7.29E-01	6.42E-01	2.23E-01	1.28E-01	2.66E-01
rs2844503	31.4	HISP	**4.66E-04**	9.03E-01	2.83E-01	2.32E-01	4.10E-01	2.13E-01
rs2516423	31.4	AA	3.37E-01	6.59E-01	5.09E-01	9.11E-01	3.11E-01	**2.11E-02**
rs371143509	31.5	HISP	2.43E-01	7.35E-01	8.40E-01	4.29E-01	1.81E-01	4.10E-01
rs3021302	32.6	HISP	**2.95E-02**	3.86E-01	**2.76E-02**	3.10E-01	5.86E-01	4.50E-01
rs28371315	32.7	AA	3.05E-01	8.69E-01	1.11E-01	2.10E-01	8.20E-01	1.84E-01
rs112394499	33.1	HISP	4.07E-01	6.14E-01	1.05E-01	2.17E-01	6.13E-01	6.18E-01

Population indicates the sample in which the variant was identified.

‡ indicates a novel variant in African Americans † indicates a novel variant in Hispanics.

**Bold** indicates p ≤ 0.05.

**Table 4:** Sex stratification.

		Females				Males			
Variant	Pop	Freq Case	Freq Control	OR (L95-U95)	P	Freq Case	Freq Control	OR (L95-U95)	P
A*02:*01*	HISP	0.14	0.20	0.68 (0.56–0.83)	**1.09E-04**	0.13	0.19	0.54 (0.10–2.75)	4.55E-01
	AA*	0.10	0.14	0.63 (0.51–0.78)	**1.21E-05**	0.10	0.10	1.05 (0.72–1.53)	7.96E-01
DQB1*02:*01*	HISP*	0.23	0.20	1.24 (1.04–1.48)	**1.72E-02**	0.19	0.22	0.91 (0.71–1.17)	4.62E-01
	AA	0.23	0.22	1.11 (0.95–1.30)	1.92E-01	0.21	0.23	0.90 (0.67–1.20)	4.57E-01
rs2516423	HISP	0.46	0.50	0.79 (0.63–1.00)	5.31E-02	0.46	0.47	0.96 (0.71–1.29)	7.66E-01
	AA*	0.49	0.49	0.89 (0.72–1.09)	2.54E-01	0.46	0.48	0.55 (0.37–0.80)	**1.82E-03**

*Indicates the sample in which the sex interaction was identified

**Bold** indicates p ≤ 0.05

### Local ancestry across the MHC

**Fig 3:**
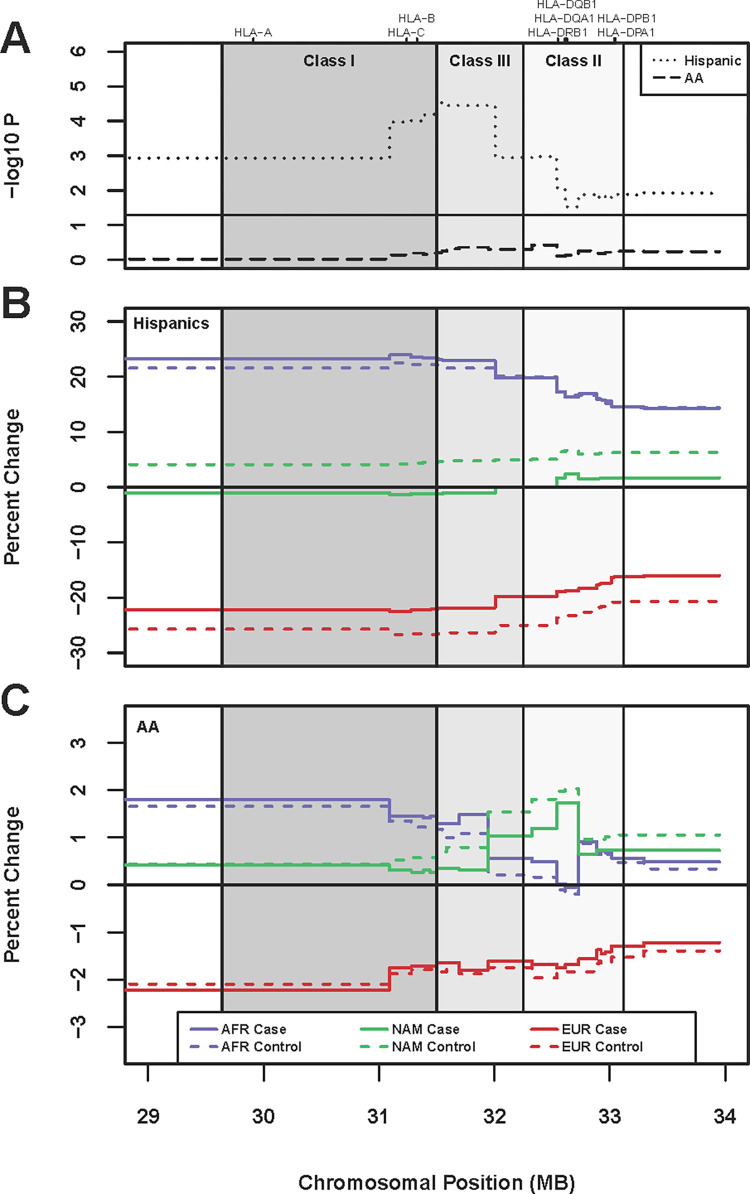
Local ancestry across the MHC. **A. Association with MS case status.** p-values from the likelihood ratio test for the effect of local ancestry on MS case status are depicted on the Y-axis and position in MB (HG19) on the x-axis. We find association of local ancestry with MS case status (p < 0.05, i.e. -log10P = 1.3) across the entire MHC in Hispanics, but no significance is observed at the same threshold in African Americans. **B. Average percent change in local vs global ancestry for Hispanic cases and controls.** An increase in African local ancestry and a decrease in European local ancestry relative to global ancestry is observed in both MS cases and controls across the entire region, consistent with admixture-enabled selection [31]. On average, Hispanic controls exhibit a greater decrease in European local ancestry relative to global ancestry than MS cases, in conjunction with a marked increase in Native American local ancestry. Differences peak between the class I and class III gene region, corresponding to the increase in -log10P denoted in (A). **C. Average percent change in local vs global ancestry for African American cases and controls.** Consistent with Hispanics, we observe an increase in African local ancestry and a decrease in European local ancestry relative to global ancestry in MS cases and controls across the entire region. The changes are of consistent magnitude in MS cases and controls, except for ~31 to ~32.6 MB between the class I and class II gene regions, where we see on average that African American controls exhibit a lesser increase in African local ancestry relative to global ancestry than MS cases, again in conjunction with a marked increase in Native American local ancestry (although -log10P does not reach a threshold for significance).

Within the Hispanic sample, we found an association of local ancestry with MS risk across the entire MHC from 29 to 34 MB (LRT p < 0.05), with ancestral differences between MS cases and controls peaking between the Class I and Class III gene regions (minimum observed LRT p = 2.99 x 10^−05^ from 31.51 to 31.56 MB, Fig 3A). In accordance with previous reports in the literature of MHC-specific admixture-enabled selection due to rapid adaptive evolution [31], relative to global ancestry, we observed an increase in local African ancestry and a decrease in local European ancestry for both MS cases and controls across the entirety of the MHC (Fig 3B). However, the magnitude of the divergence of local ancestry from global ancestry differs by MS status. Relative to controls, MS cases had an increase in both EUR and AFR ancestry (average increase of 4.5% EUR and 1% AFR in MS cases relative to controls), in conjunction with a substantial decrease in NAM ancestry (average decrease of 5.5% in MS cases relative to controls, LRT p < 0.01 from 31 to 32.6 MB).

The same increase in local African ancestry and decrease in local European ancestry relative to global ancestry was observed across the MHC in all African American samples. While the magnitude of divergence did not statistically differ between MS cases and controls (LRT p > 0.05), a similar pattern as had been seen in the Hispanic sample was seen beginning between the Class I and Class III gene region and extending into Class II; where relative to controls, MS cases had an increase in both EUR and AFR ancestry, in conjunction with a decrease in NAM ancestry (Fig 3C). Given the low levels of NAM ancestry in the population (~2% in MS cases and controls), the average decrease in MS cases relative to controls was modest (only ~0.4% across this region from ~31 to 32.6 MB as compared to 5.5% in Hispanics); nonetheless, the pattern is strikingly consistent.

### Global ancestry modification of MS risk

Two of the twelve independent MHC variants identified for association with MS in the Hispanic sample (Table 1) demonstrated global ancestry risk modification (p < 0.05) (Table 3); rs2844503 (additive OR = 1.34 in the full sample), an intergenic variant flanking *MICB-DT*, and rs3021302 (recessive OR = 3.18 in the full sample), an intergenic variant flanking *HLA-DQB1*.

**Fig 4:**
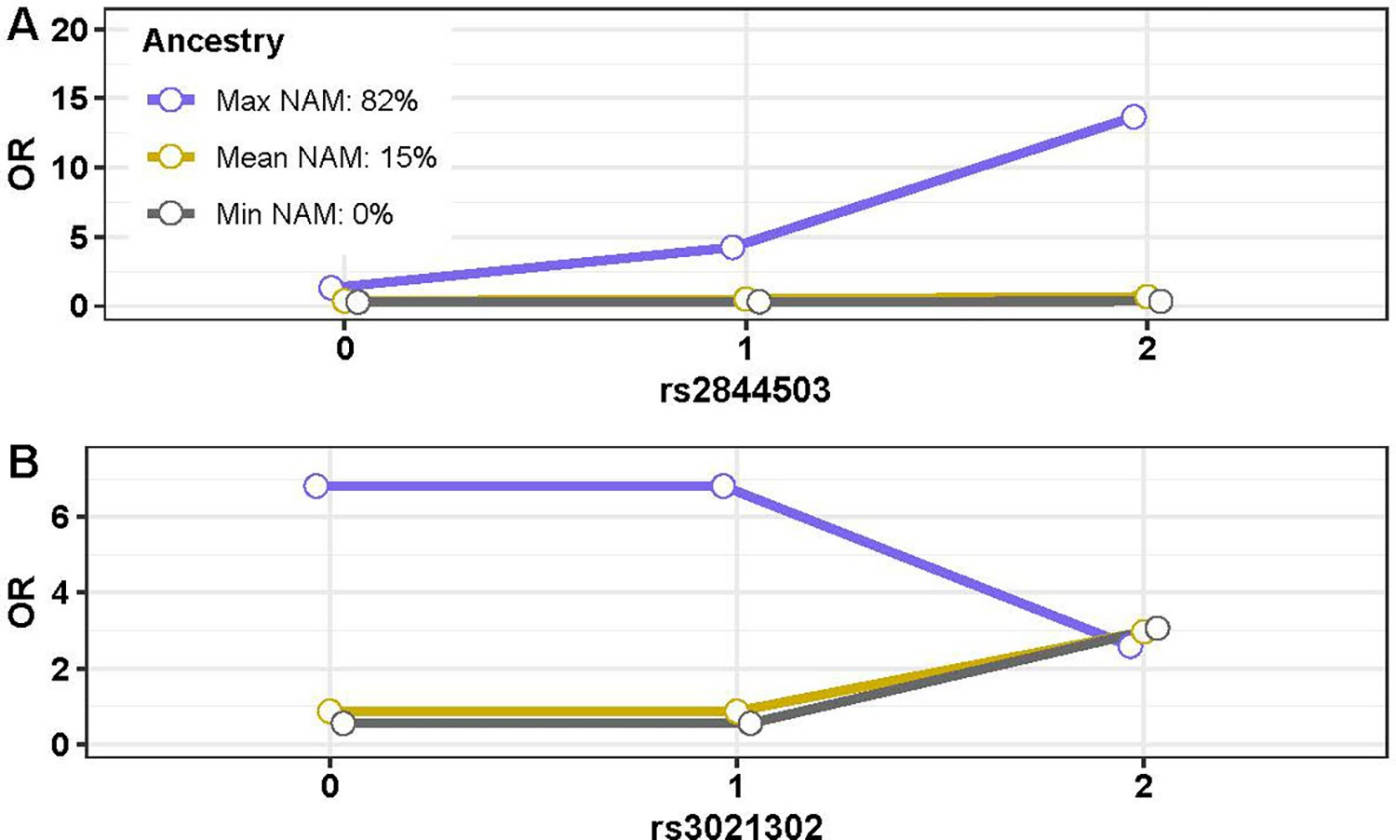
Global ancestry risk modification in Hispanics. Expected odds ratios (OR) are depicted on the Y axis for individuals with 0, 1, or 2 copies of the variant allele for each SNP, under varying levels of NAM ancestry. **A.** rs2844503 demonstrates a risk effect in individuals with the maximum observed NAM while no effect is seen for individuals with the minimum or mean observed NAM. **B.** rs3021302 demonstrates a protective effect in individuals with the maximum observed NAM while a risk effect is seen in individuals with the minimum or mean observed NAM.

To investigate if the degree of MS risk attributed to the variant allele was dependent upon global ancestry composition, we estimated the odds of MS under various ancestral scenarios. For rs2844503, we first considered an individual with no NAM ancestry, and we estimated odds of MS to be 0.29, 0.32, and 0.35 for each of 0, 1, or 2 observed variant alleles utilizing the parameter estimates obtained in Eq (3). We then considered an individual with 15% NAM ancestry (the mean observed in our sample) and estimated odds of MS to be 0.38, 0.51, and 0.58 for each of 0, 1, or 2 observed variant alleles. Conversely, the estimated odds of MS increased exponentially with each allelic copy for an individual with 82% NAM ancestry (the maximum observed in our sample); with estimated odds of MS being 1.32, 4.25, and 13.67 for 0, 1, or 2 observed variant alleles. Thus, we conclude that variation in rs2844503 presents a risk for MS primarily in individuals with a high proportion of NAM ancestry (Fig 4A). While individual global ancestry drastically alters the effect of rs2844503 on MS risk, we found that individual local ancestry within the specified genomic region did not demonstrate risk modification (Table 3).

For rs3021302, we found that estimated MS risk decreased recessively for an individual with 82% NAM ancestry. The estimated odds of MS was 6.82 for zero or one variant allele copy and 2.58 for two copies. Conversely, estimated MS risk increased recessively for an individual with the no NAM ancestry (estimated odds of MS was 0.54 for zero or one allele copy and 3.06 for two copies) and 15% NAM ancestry (estimated odds of MS was 0.86 for zero or one allele copy and 2.97 for two copies). We conclude that variation in rs3021302 presents a risk for MS primarily in individuals with a low proportion of NAM ancestry and confers a protective effect in individuals with a high proportion of NAM ancestry (Fig 4B). An interaction between local ancestry and rs3021302 was also observed (Table 3).

**Fig 5:**
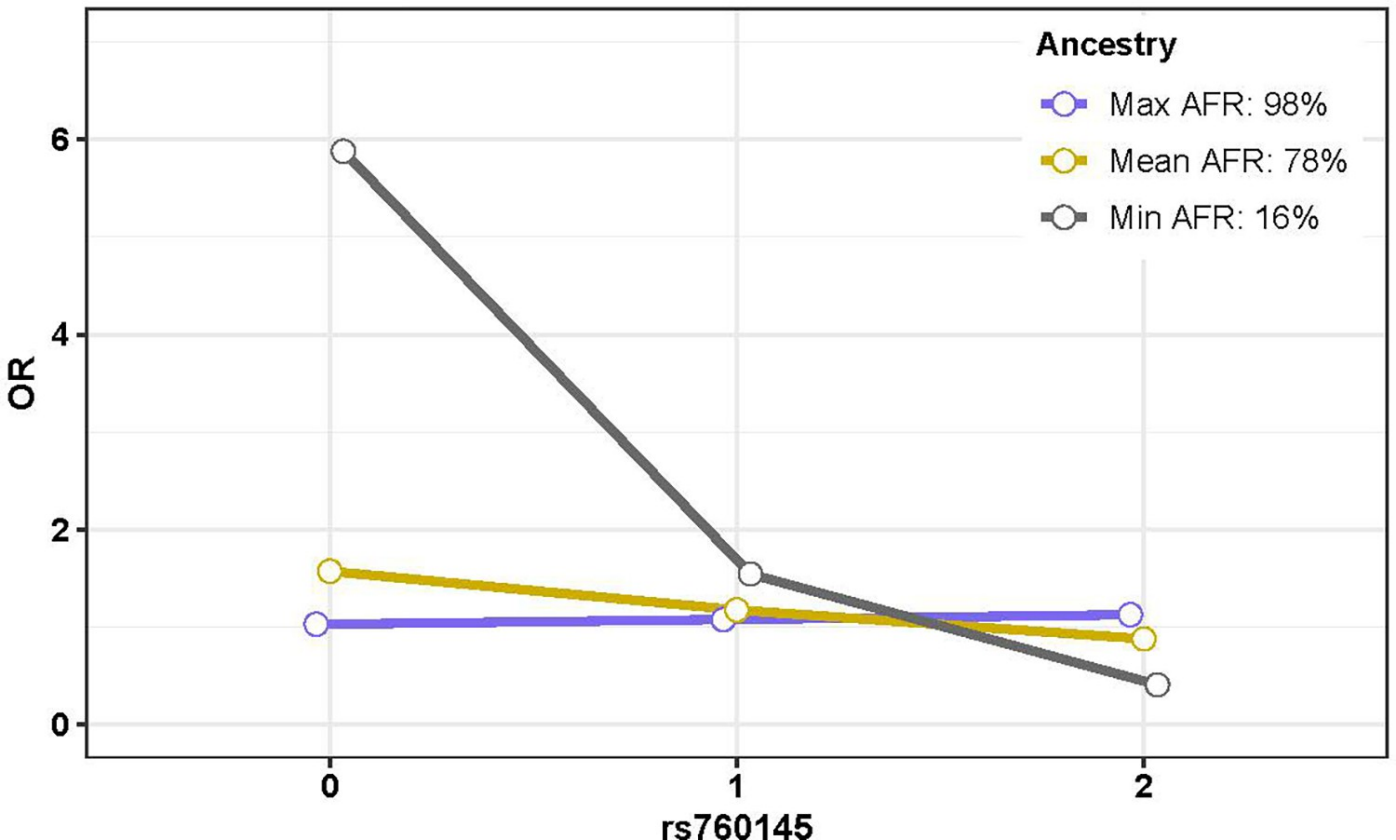
Global ancestry risk modification in African Americans. Expected odds ratios (OR) are depicted on the Y axis for individuals with 0, 1, or 2 copies of the variant allele for each SNP, under varying levels of AFR ancestry. A protective effect is seen in individuals with the minimum observed AFR while no effect is seen for individuals with the maximum or mean observed AFR.

One of the six independent MHC variants identified for association with MS in the African American sample (Table 2) demonstrated global ancestry risk modification (Table 3); rs760145 (additive OR = 0.75 in the full sample), an intronic variant in *HLA-F-AS1*, which also represents a novel signal in African Americans. The estimated risk of MS decreased exponentially with each allelic copy for an individual with the 16% AFR ancestry (the minimum observed in our sample), with estimated odds of MS being 5.88, 1.54, and 0.40 for 0, 1, and 2 observed variant alleles (Fig 5). A more moderate decrease in risk was seen for an individual with 78% AFR ancestry (the mean observed in our sample), with estimated odds of MS being 1.57, 1.17, and 0.87 for 0, 1, and 2 observed variant alleles. In contrast, a slight increase was observed for an individual with 98% AFR ancestry (the maximum observed in our sample), with estimated odds of MS being 1.02, 1.07, and 1.12 for 0, 1, and 2 observed variant alleles. We conclude that the protective effect of variation in rs760145 occurs in individuals of low AFR ancestry, with minimal effect seen for those with high levels of AFR ancestry. There was no significant interaction between local ancestry and rs760145 (Table 3).

### Local ancestry modification of MS risk

Local ancestry risk modification (p < 0.05) was observed for four of the MS associated classical alleles (HLA-A*02:*01*, HLA-B*53:*01*, HLA-DRB1*15:*01*, and HLA-DQB1*06:*02*) and two SNPs (rs6929950 and rs3021302) (Table 3). While statistical significance of the interaction between local ancestry and allele / SNP may have only been observed in either the Hispanic or African American samples, apart from HLA-DQB1*06:*02* where interaction was observed in both (Table 3), a haplotype model was used to assess allele specific association, stratified by ancestral allele origin in both samples to determine consistency in direction of ancestral effect (Table 5). Given the recessive nature of rs3021302, a haplotypic model was not applied.

**Table 5:** Local ancestry stratification.

		EUR Alleles			AFR Alleles			NAM Alleles		
Variant	Pop	Case / Control	OR (L95-U95)	P	Case / Control	OR (L95-U95)	P	Case / Control	OR (L95-U95)	P
A*02:*01*	HISP	244/385	0.63 (0.52–075)	**7.44E-07**	34/43	0.80 (0.50–1.28)	3.61E-01	69/67	1.06 (0.73–1.54)	7.57E-01
	AA*	109/135	0.56 (0.42–0.76)	**1.73E-04**	136/151	0.77 (0.60–0.98)	**3.06E-02**	10/3	NA	NA
B*53:*01*	HISP*	3/2	NA	NA	42/61	0.66 (0.44–1.01)	5.72E-02	0/0	NA	NA
	AA	1/0	NA	NA	232/276	0.71 (0.59–0.86)	**3.42E-04**	0/1	NA	NA
DRB1*15:*01*	HISP	376/181	2.67 (2.19–3.26)	**4.49E-22**	4/2	NA	NA	0/0	NA	NA
	AA*	143/70	2.02 (1.45–2.80)	**2.68E-05**	5/1	NA	NA	0/0	NA	NA
DQB1*06:*02*	HISP*	363/163	2.90 (2.36–3.56)	**6.22E-24**	72/56	1.36 (0.93–1.98)	1.09E-01	0/0	NA	NA
	AA*	167/79	2.11 (1.55–2.89)	**2.34E-06**	465/383	1.11 (0.95–1.29)	1.96E-01	0/0	NA	NA
rs6929950	HISP	11/30	0.37 (0.18–0.74)	**7.38E-03**	39/85	0.44 (0.30–0.66)	**5.87E-05**	0/1	NA	NA
	AA*	6/6	0.86 (0.27–2.70)	7.96E-01	203/212	0.84 (0.69–1.03)	9.44E-02	0/5	NA	NA

* Indicates the sample in which the local ancestry interaction was identified

Case / Control indicates the number of ancestral alleles observed for each phenotypic state

Stratified analysis not conducted if the number of population specific alleles < 15

**Bold** indicates p ≤ 0.05

For HLA-A*02:*01*, while the significant local ancestry interaction was observed in the African American sample (p = 4.14 x10^-02^), both the Hispanic and the African American sample demonstrate a stronger protective effect on MS for ancestral EUR alleles than for AFR alleles (EUR OR = 0.63, AFR OR = 0.80 in Hispanics; EUR OR = 0.56, AFR OR = 0.77 in African Americans). Interestingly, there are a greater number of NAM case alleles than control alleles in both samples; although the difference is non-significant, indicating a risk effect for HLA-A*02:*01* alleles of NAM ancestral descent (Table 5).

For HLA-B*53:*01*, a local ancestry interaction was observed in Hispanics (p = 4.37 x 10^−02^) and nominally in African Americans (p = 7.02 x 10^−02^) (Table 3). While HLA-B*53:*01* demonstrated a significant protective effect in the overall African American sample (Table 2), no association was seen with MS in the overall Hispanic sample (p = 1.12 x 10^−01^). Yet, within the Hispanic sample, a nominal protective effect was seen for HLA-B*53:*01* alleles of AFR descent (OR = 0.66; p = 5.72 x 10^−02^) (Table 5). Although the majority of HLA-B*53:*01* alleles are of AFR descent, a non-zero number of EUR and NAM alleles were observed in both Hispanics and African Americans; consistent with expected frequencies from reference populations (S4 Table). In both samples, more EUR case alleles were observed than EUR control alleles (3 vs 2 in Hispanics and 1 vs 0 in African Americans).

Both HLA-DRB1*15:*01* and HLA-DQB1*06:*02* demonstrate MS risk that is largely attributable to EUR alleles in both the Hispanic and African American samples (Table 5). For HLA-DRB1*15:*01*, fewer than 10 non-EUR alleles were observed in each sample (Table 5). Substantially more AFR alleles were observed for HLA-DQB1*06:*02* than for HLA-DRB1*15:*01*; however, the observed odds ratios (OR) for EUR allele carriers is almost two times that of AFR allele carriers with non-overlapping confidence intervals (CI) between ancestral allele origins, indicating a statistical difference in effect (EUR OR = 2.90 with CI: 2.36–3.56, AFR OR = 1.36 with CI: 0.93–1.98 in Hispanics; EUR OR = 2.11 with CI: 1.55–2.89, AFR OR = 1.11 with CI: 0.95–1.29 in African Americans).

Lastly, a significant local-ancestry interaction was identified in the African American sample for rs6929950, an intronic variant within *OR5V1* (Table 5). Although this variant was identified as a novel protective variant for MS in the Hispanic sample (Table 1), marginal association was also observed in the African American sample (OR = 0.82, p = 4.97 x 10^−02^). In the African American sample, most observed alleles are of AFR descent (OR = 0.84, p = 9.44 x 10^−02^). Only 12 observed alleles are of EUR descent (6 in MS cases and 6 in controls, demonstrating no effect), and 5 observed alleles are of NAM descent (all 5 in controls, demonstrating a highly protective effect). The small non-AFR sample size and lack of effect observed for EUR alleles is likely driving the observed local ancestry interaction, but larger samples would be needed to determine validity. Conversely, no real difference in effect is seen between EUR (OR = 0.37) and AFR (OR = 0.44) ancestral alleles in Hispanics.

## Discussion

In this large MS multiethnic cohort we have identified an independent contribution of HLA-DRB1*15:*01* and HLA-DQB1*06:*02* to MS risk in both the Hispanic and African American sample. We have additionally identified a striking decrease in NAM ancestry in cases relative to controls across the MHC which can be seen in both Hispanics and African Americans, peaking between the Class I and Class III gene region. We found several MS susceptibility variants to have an effect that is modified by global ancestry; indicating ancestral differences which may be due in part to correlated socio-economic or environmental factors, and we have also identified MS susceptibility variants which have an effect that is modified by local ancestry, indicating true genetic differences in the degree of risk/protection exerted across ancestral backgrounds. We have discovered several novel susceptibility variants, and confirmed robust replication (p < 1 × 10^−03^) for six classical alleles in Hispanics and two classical alleles in African Americans which had been previously identified in Europeans.

Despite our Hispanic and African American study samples being similar in size, we have observed substantially more replication of previously identified alleles in Hispanics at the specified significance level. This contrasts with another study of the MHC in Hispanics and African Americans conducted by Chi et al. which reported association with MS at the same significance level for only HLA-DRB1*15:*01* in Hispanics [12] and HLA-DRB1*15:*01* and HLA-DRB1*03:*01* in African Americans. At a broader level, they identified more replication of classical alleles at p < 0.05 in African Americans than Hispanics and concluded that there may be a smaller overlap in MHC specific MS genetic risk between Hispanics and Europeans than that of African Americans and Europeans. This contrast could be due in part to their relatively smaller Hispanic case collection (326 Hispanic MS cases) as well as differences in Hispanic ancestral background between the studies. Chi et al. have reported considerably more Native American (average 34% vs 18% among MS cases) and less European ancestry (average 56% vs 71% among MS cases) than our sample [12].

Using stepwise conditional modeling we found that HLA-DQB1*06:*02* was significantly associated with MS after conditioning on HLA-DRB1*15:*01* in African Americans and vice versa in Hispanics. This provides evidence that in admixed populations, HLA-DRB1*15:*01* and HLA-DQB1*06:*02* contribute to MS risk in a manner that is independent of one another. Within European populations, HLA-DRB1*15:*01* is most often found as part of an extended haplotype with HLA-DQB1*06:*02*, and a decade of fine-mapping research has sought to distinguish which is the predisposing allele [32]. One of the largest studies in Europeans concluded that the association signal could be localized to HLA-DRB1*15:*01* [6]. A similar study in African Americans, containing a small subset of the samples in the current study, also identified HLA-DRB1*15:*01* as the predominant signal, finding no effect of HLA-DQB1*06:*02* in the absence of HLA-DRB1*15:*01*; however, the study included only ~350 patients with MS and ~300 controls, and less than 25 individuals were identified as being HLA-DRB1*15:*01-* and HLA-DQB1*06:*02+* in either the patient or control cohort [11]. Within our larger study samples, we do however observe an effect of HLA-DQB1*06:*02* in the absence of HLA-DRB1*15:*01*, in both Hispanics and African Americans (p < 0.05 for both populations, S5 Table), consistent with our determination of independence from the conditional model. Although an independent association for HLA-DRB1*15:*01* and HLA-DQB1*06:*02* has been undetectable in Europeans due to high LD, it is possible that a European model for biologically independent contributions also exists.

A striking decrease of NAM ancestry in MS Hispanic cases relative to controls across the extended MHC was found. This suggests that protective NAM haplotypes are likely present across the region. These differences peak between the Class I and Class III gene region and are centered on the previously identified *MICB*/*LST1* locus. While the magnitude of this difference is substantially smaller in African Americans, a similar pattern is observed. Further work is needed to understand the role this difference plays in variation in both disease incidence and disease presentation between the two populations. Minimal effort has been made to fine-map the previously identified *MICB/LST1* locus in European populations [6], and the signal has not yet been refined. It is possible that Hispanic or indigenous populations with a substantial NAM component may be most advantageous for fine-mapping of the locus. Notably, within this region we also identified three independent signals in Hispanics (with one, rs2844503, demonstrating NAM global ancestry risk modification in the absence of a local ancestry modification) and one in African Americans, all of which were in low LD (R2 ≤ 0.2) with the variants previously identified in European populations. This may indicate that substantial locus heterogeneity is also present within this region, with the additional presence of variants modified by global ancestry indicating that environmental factors may also play a role in the influence of the *MICB* locus on MS susceptibility.

We identified three novel protective variants for MS across the extended MHC, one in the Hispanic sample (rs6929950, an intronic variant within *OR5V1*) and two in the African American sample (classical HLA-B*53:*01* and rs760145, an intronic variant within *HLA-F-AS1*). While rs6929950 was detected at the specified significance threshold (p < 1.00 x 10^−03^) in Hispanics and demonstrated only nominal association in African Americans (p = 4.97 x 10^−02^), more than 75% of the variant alleles identified in both samples were of African origin. It is worthwhile to note that local ancestry risk modification was detected for both rs6929950 and HLA-B*53:*01;* although in both instances the frequency of non-African alleles detected was low, with ≤5 for HLA-B*53:*01* and ≤20 for rs6929950, necessitating that local ancestry interactions be interpreted with caution. Variant rs6929950, located within *OR5V1*, has regulatory potential, demonstrating a regulomeDB score of (3a) [33] indicative of its location within a transcription factor binding site and DNase peak. Although further refinement would be needed to attribute causality of the variant, *OR5V1* represents an important biological candidate for MS susceptibility given the notable olfactory dysfunction among a number of neurodegenerative diseases [34]. HLA-B*53:*01* also constitutes an important novel mechanism for MS protection which may be unique to individuals of African descent. It was the first HLA allele to be associated with resistance to severe malaria [35] and is found in 12% of African individuals while rarely occurring in other populations (S4 Table), suggestive of positive selection.

The third novel variant, intronic rs760145 in *HLA-F-AS1* demonstrates differing effect allele frequencies by ancestry, being 0.435, 0.532, and 0.602 in gnomAD Africans, Europeans, and Americas populations respectively (S4 Table). Global ancestry modification is also present, while no local ancestry modification is seen, indicating that these effect differences may represent complex socio-economic or other environmental interactions. While there has been some previous evidence for association of variation within *HLA-F-AS1* and MS [36], moderate LD exists between *HLA-A*:*02*:*01* and rs760145 in Europeans (R2 = 0.2) while LD is negligible in African Americans and may explain why *HLA-F-AS1* has not been indicated as an independent MS locus in more recent studies [1].

We did not find significance for the previously identified African allele, HLA-DRB1*15:*03* at the pre-specified significance level of 1.0 x 10^−04^; however, after conditioning on the three independent African classical alleles, marginal significance was seen (OR = 1.23, p = 2.53 x 10^−02^), indicating that HLA-DRB1*15:*03* does contribute to MS risk within this sample. Marginal significance was eliminated after conditioning on intergenic rs28371315; pointing to the extended haplotypes that exist between *DRB1* and *DQB1*.

Local ancestry risk modification was seen for several well-established MS susceptibility alleles including HLA-DRB1*15:*01*, HLA-DQB1*06:*02*, and HLA-A*02:*01*. Given that these were among the first alleles identified in Europeans and have been consistently and robustly replicated, it is perhaps unsurprising that all three alleles demonstrate effects which are largely augmented on the EUR ancestral haplotype. Most notably, for HLA-DQB1*06:*02*, we find MS risk for EUR allele carriers is almost two times the risk for AFR allele carriers. Although HLA-DQB1*06:*02* did not pass quality control thresholds in their study, a similar pattern for HLA-DRB1*15:*01* was identified by Chi et al. who determined that risk of MS conferred by the EUR HLA-DRB1*15:*01* allele was three times higher compared to the AFR HLA-DRB1*15:*01* allele. In our study, less than 10 non-European HLA-DRB1*15:*01* alleles were identified in the Hispanic or African American sample. Although a local ancestry interaction was observed, not much inference can be taken from the ancestral stratified analyses.

Our sample represents the largest and most geographically diverse collection of Hispanic and African American individuals with MS to date. However, we acknowledge that our sample size is still limited and thus there are likely additional novel and previously identified MHC associations that have gone undetected in our sample. Similarly, detection of effects which are heterogeneous by sex or ancestral state may be limited, and false positive interactions may remain unidentified. Additionally, there is the possibility that we may be missing an extended haplotype with a causal *DRB1* allele which is linked to DQB1*06:*02* but has gone undetected due to power [37]. Nonetheless, a previous study in the isolated founder population of Sardinia also identified an independent association for the HLA-DRB1*15:*01* and HLA-DQB1*06:*02* alleles [38]; a suggestion which has also been supported in transgenic mouse models [39–41].

In conclusion, we observe a central role for ancestry in genetic risk modification across the MHC. Both global and local variant-specific ancestral risk modifications have been identified which may influence prevalence or phenotypic differences that have been observed across racial and ethnic groups [42–44]. More broadly, we have observed a decrease in Native American ancestry in MS cases relative to controls across much of the extended MHC, most notably among Hispanics, indicating that protective Native American haplotypes are likely present across much of the region. We have identified several MHC-specific MS susceptibility variants in the admixed African American and Hispanic samples which are rare in European populations and represent novel population-specific effects, and we have also utilized the differing LD patterns in Hispanics and African Americans to confirm an independent role for HLA-DRB1*15:*01* and HLA-DQB1*06:*02* on MS risk. These results taken together validate the importance of investigating MS susceptibility at an ancestral level in heterogeneous populations to identify population-specific genetic influence on disease risk and offer insight into the epidemiology of MS phenotypic diversity.

The current study focused on imputed genomic array data within the MHC containing rich information in genetic ancestry, population genomics, and MS susceptibility. Future research can integrate additional -omics data; including but not limited to transcriptomics, epigenomics, proteomics, and pharmacogenomics to further decipher the relationship between genetic ancestry and MS susceptibility within the MHC and within other known MS loci [45]; facilitating a comprehensive understanding of precision population health.

## Supporting information

S1 Table Imputation quality assessment. (XLSX)

S2 Table Marginal additive association of classical HLA alleles in Hispanics. (XLSX)

S3 Table Marginal additive association of classical HLA alleles in African Americans. (XLSX)

S4 Table Frequencies of independent MHC variants in diverse populations. (XLSX)

S5 Table DRB1*15:01 and DQB1*06:02 genotypes in Hispanics and African Americans. (XLSX)

## References

[1] International Multiple Sclerosis Genetics Consortium (IMSGC). Multiple Sclerosis Genomic Map implicates peripheral immune cells and microglia in susceptibility. Science 2019;365(6460). 10.1126/science.aav7188 PMC7241648 31604244

[2] Jersild C, Fog T, Hansen GS, Thomsen M, Svejgaard A, Dupont B. Histocompatibility determinants in multiple sclerosis, with special reference to clinical course. Lancet 1973 Dec 1;2(7840):1221–1225. 4128558 10.1016/s0140-6736(73)90970-7

[3] Compston D, Batchelor J, McDonald W. B-lymphocyte alloantigens associated with multiple sclerosis. The Lancet 1976;308(7998):1261–1265. 10.1016/s0140-6736(76)92027-4 63743

[4] Isobe N, Madireddy L, Khankhanian P, Matsushita T, Caillier SJ, More JM, et al. An ImmunoChip study of multiple sclerosis risk in African Americans. Brain 2015 Jun;138(Pt 6):1518–1530. 25818868 10.1093/brain/awv078 PMC4553906

[5] International Multiple Sclerosis Genetics Consortium, Wellcome Trust Case Control Consortium 2, Sawcer S, Hellenthal G, Pirinen M, Spencer CC, et al. Genetic risk and a primary role for cell-mediated immune mechanisms in multiple sclerosis. Nature 2011 Aug 10;476(7359):214–219. 21833088 10.1038/nature10251 PMC3182531

[6] Patsopoulos NA, Barcellos LF, Hintzen RQ, Schaefer C, van Duijn CM, Noble JA, et al. Fine-mapping the genetic association of the major histocompatibility complex in multiple sclerosis: HLA and non-HLA effects. PLoS Genet 2013 Nov;9(11):e1003926. 24278027 10.1371/journal.pgen.1003926 PMC3836799

[7] Moutsianas L, Jostins L, Beecham AH, Dilthey AT, Xifara DK, Ban M, et al. Class II HLA interactions modulate genetic risk for multiple sclerosis. Nat Genet 2015 Oct;47(10):1107–1113. 26343388 10.1038/ng.3395 PMC4874245

[8] Ramagopalan SV, Ebers GC. Epistasis: multiple sclerosis and the major histocompatibility complex. Neurology 2009 Feb 10;72(6):566–567. 19204267 10.1212/01.wnl.0000341941.24967.e6 PMC2818182

[9] Ramagopalan SV, Maugeri NJ, Handunnetthi L, Lincoln MR, Orton SM, Dyment DA, et al. Expression of the multiple sclerosis-associated MHC class II Allele HLA-DRB1*1501 is regulated by vitamin D. PLoS Genet 2009 Feb;5(2):e1000369. 19197344 10.1371/journal.pgen.1000369 PMC2627899

[10] Murphy K. Janeway’s Immunobiology. Garland Science; 2011.

[11] Oksenberg JR, Barcellos LF, Cree BA, Baranzini SE, Bugawan TL, Khan O, et al. Mapping Multiple Sclerosis Susceptibility to the HLA-DR Locus in African Americans. Am J Hum Genet 2004 01;74:160–167. 14669136 10.1086/380997 PMC1181903

[12] Chi C, Shao X, Rhead B, Gonzales E, Smith JB, Xiang AH, et al. Admixture mapping reveals evidence of differential multiple sclerosis risk by genetic ancestry. PLoS Genet 2019 Jan 17;15(1):e1007808. 30653506 10.1371/journal.pgen.1007808 PMC6353231

[13] Rivera VM. Multiple Sclerosis in Latin Americans: Genetic Aspects. Curr Neurol Neurosci Rep 2017 Aug;17(8):57-017-0768-4. 10.1007/s11910-017-0768-4 28639238

[14] Alaez C, Corona T, Ruano L, Flores H, Loyola M, Gorodezky C. Mediterranean and Amerindian MHC class II alleles are associated with multiple sclerosis in Mexicans. Acta Neurol Scand 2005 Nov;112(5):317–322. 16218914 10.1111/j.1600-0404.2005.00493.x

[15] Brum DG, Barreira AA, Louzada-Junior P, Mendes-Junior CT, Donadi EA. Association of the HLA-DRB1*15 allele group and the DRB1*1501 and DRB1*1503 alleles with multiple sclerosis in White and Mulatto samples from Brazil. J Neuroimmunol 2007 Sep;189(1–2):118–124. 17681614 10.1016/j.jneuroim.2007.06.009

[16] Silva AM, Pereira C, Bettencourt A, Carvalho C, Couto AR, Leite MI, et al. The role of HLA-DRB1 alleles on susceptibility and outcome of a Portuguese Multiple Sclerosis population. J Neurol Sci 2007 Jul 15;258(1–2):69–74. 17412364 10.1016/j.jns.2007.02.033

[17] Alves-Leon SV, Papais-Alvarenga R, Magalhaes M, Alvarenga M, Thuler LC, Fernandez y Fernandez O. Ethnicity-dependent association of HLA DRB1-DQA1-DQB1 alleles in Brazilian multiple sclerosis patients. Acta Neurol Scand 2007 May;115(5):306–311. 17489940 10.1111/j.1600-0404.2006.00750.x

[18] Rojas OL, Rojas-Villarraga A, Cruz-Tapias P, Sanchez JL, Suarez-Escudero JC, Patarroyo MA, et al. HLA class II polymorphism in Latin American patients with multiple sclerosis. Autoimmun Rev 2010 Apr;9(6):407–413. 19896562 10.1016/j.autrev.2009.11.001

[19] Khan O, Williams MJ, Amezcua L, Javed A, Larsen KE, Smrtka JM. Multiple sclerosis in US minority populations: Clinical practice insights. Neurol Clin Pract 2015 Apr;5(2):132–142. 26137421 10.1212/CPJ.0000000000000112 PMC4404283

[20] Beecham AH, Amezcua L, Chinea A, Manrique CP, Rubi C, Isobe N, et al. The genetic diversity of multiple sclerosis risk among Hispanic and African American populations living in the United States. Mult Scler 2019 Aug 1:1352458519863764. 10.1177/1352458519863764 PMC6994382 31368393

[21] 1000 Genomes Project Consortium, Auton A, Brooks LD, Durbin RM, Garrison EP, Kang HM, et al. A global reference for human genetic variation. Nature 2015 Oct 1;526(7571):68–74. 26432245 10.1038/nature15393 PMC4750478

[22] Zheng X, Shen J, Cox C, Wakefield JC, Ehm MG, Nelson MR, et al. HIBAG—HLA genotype imputation with attribute bagging. Pharmacogenomics J 2014 Apr;14(2):192–200. 23712092 10.1038/tpj.2013.18 PMC3772955

[23] Wojcik G, Graff M, Nishimura KK, Tao R, Haessler J, Gignoux CR, et al. The PAGE Study: How Genetic Diversity Improves Our Understanding of the Architecture of Complex Traits. bioRxiv 2018(188094). http://biorxiv.org/content/early/2018/10/17/188094.abstract.

[24] Gourraud PA, Khankhanian P, Cereb N, Yang SY, Feolo M, Maiers M, et al. HLA diversity in the 1000 genomes dataset. PLoS One 2014 Jul 2;9(7):e97282. 24988075 10.1371/journal.pone.0097282 PMC4079705

[25] Maples BK, Gravel S, Kenny EE, Bustamante CD. RFMix: a discriminative modeling approach for rapid and robust local-ancestry inference. Am J Hum Genet 2013 Aug 8;93(2):278–288. doi: 10.1016/j.ajhg.2013.06.020 23910464 PMC3738819

[26] Alexander DH, Novembre J, Lange K. Fast model-based estimation of ancestry in unrelated individuals. Genome Res 2009 Sep;19(9):1655–1664. doi: 10.1101/gr.094052.109 19648217 PMC2752134

[27] Bryc K, Durand EY, Macpherson JM, Reich D, Mountain JL. The genetic ancestry of African Americans, Latinos, and European Americans across the United States. Am J Hum Genet 2015 Jan 8;96(1):37–53. 25529636 10.1016/j.ajhg.2014.11.010 PMC4289685

[28] Hensiek AE, Sawcer SJ, Feakes R, Deans J, Mander A, Akesson E, et al. HLA-DR 15 is associated with female sex and younger age at diagnosis in multiple sclerosis. J Neurol Neurosurg Psychiatry 2002 02;72:184–187. doi: 10.1136/jnnp.72.2.184 11796767 PMC1737743

[29] Barcellos LF, Sawcer S, Ramsay PP, Baranzini SE, Thomson G, Briggs F, et al. Heterogeneity at the HLA-DRB1 locus and risk for multiple sclerosis. Hum Mol Genet 2006 09/15;15(1):2813–2824. doi: 10.1093/hmg/ddl223 16905561

[30] Irizar H, Munoz-Culla M, Zuriarrain O, Goyenechea E, Castillo-Trivino T, Prada A, et al. HLA-DRB1*15:01 and multiple sclerosis: a female association? Mult Scler 2012 May;18(5):569–577. 22127897 10.1177/1352458511426813

[31] Norris ET, Rishishwar L, Chande AT, Conley AB, Ye K, Valderrama-Aguirre A, et al. Admixture-enabled selection for rapid adaptive evolution in the Americas. Genome Biol 2020 Feb 7;21(1):29-020-1946-2. doi: 10.1186/s13059-020-1946-2 PMC7006128 32028992

[32] Hollenbach JA, Oksenberg JR. The immunogenetics of multiple sclerosis: A comprehensive review. J Autoimmun 2015 Nov;64:13–25. 26142251 10.1016/j.jaut.2015.06.010 PMC4687745

[33] Boyle AP, Hong EL, Hariharan M, Cheng Y, Schaub MA, Kasowski M, et al. Annotation of functional variation in personal genomes using RegulomeDB. Genome Res 2012 Sep;22(9):1790–1797. 22955989 10.1101/gr.137323.112 PMC3431494

[34] Barresi M, Ciurleo R, Giacoppo S, Foti Cuzzola V, Celi D, Bramanti P, et al. Evaluation of olfactory dysfunction in neurodegenerative diseases. J Neurol Sci 2012 Dec 15;323(1–2):16–24. 23010543 10.1016/j.jns.2012.08.028

[35] Hill AV, Allsopp CE, Kwiatkowski D, Anstey NM, Twumasi P, Rowe PA, et al. Common west African HLA antigens are associated with protection from severe malaria. Nature 1991 Aug 15;352(6336):595–600. 1865923 10.1038/352595a0

[36] De Jager PL, Jia X, Wang J, de Bakker PI, Ottoboni L, Aggarwal NT, et al. Meta-analysis of genome scans and replication identify CD6, IRF8 and TNFRSF1A as new multiple sclerosis susceptibility loci. Nat Genet 2009 Jul;41(7):776–782. 19525953 10.1038/ng.401 PMC2757648

[37] Creary LE, Mallempati KC, Gangavarapu S, Caillier SJ, Oksenberg JR, Fernandez-Vina MA. Deconstruction of HLA-DRB1*04:01:01 and HLA-DRB1*15:01:01 class II haplotypes using next-generation sequencing in European-Americans with multiple sclerosis. Mult Scler 2019 May;25(6):772–782. 29683085 10.1177/1352458518770019 PMC6365219

[38] Marrosu MG, Murru R, Murru MR, Costa G, Zavattari P, Whalen M, et al. Dissection of the HLA association with multiple sclerosis in the founder isolated population of Sardinia. Hum Mol Genet 2001 12/01;10(2):2907–2916. 11741834 10.1093/hmg/10.25.2907

[39] Kaushansky N, Altmann DM, Ascough S, David CS, Lassmann H, Ben-Nun A. HLA-DQB1*0602 determines disease susceptibility in a new "humanized" multiple sclerosis model in HLA-DR15 (DRB1*1501;DQB1*0602) transgenic mice. J Immunol 2009 Sep 1;183(5):3531–3541. 19648275 10.4049/jimmunol.0900784

[40] Kaushansky N, Altmann DM, David CS, Lassmann H, Ben-Nun A. DQB1*0602 rather than DRB1*1501 confers susceptibility to multiple sclerosis-like disease induced by proteolipid protein (PLP). J Neuroinflammation 2012 Feb 8;9:29-2094-9-29. 10.1186/1742-2094-9-29 PMC3344688 22316121

[41] Kaushansky N, Ben-Nun A. DQB1*06:02-Associated Pathogenic Anti-Myelin Autoimmunity in Multiple Sclerosis-Like Disease: Potential Function of DQB1*06:02 as a Disease-Predisposing Allele. Front Oncol 2014 Oct 16;4:280. 25360418 10.3389/fonc.2014.00280 PMC4199271

[42] Buchanan RJ, Zuniga MA, Carrillo-Zuniga G, Chakravorty BJ, Tyry T, Moreau RL, et al. Comparisons of Latinos, African Americans, and Caucasians with multiple sclerosis. Ethn Dis 2010 Autumn;20(4):451–457. 21305836

[43] Amezcua L, Oksenberg JR, McCauley JL. MS in self-identified Hispanic/Latino individuals living in the US. Mult Scler J Exp Transl Clin 2017 Sep 25;3(3):2055217317725103. 28979795 10.1177/2055217317725103 PMC5617095

[44] Amezcua L, Beecham AH, Delgado SR, Chinea A, Burnett M, Manrique CP, et al. Native ancestry is associated with optic neuritis and age of onset in hispanics with multiple sclerosis. Ann Clin Transl Neurol 2018 Sep 23;5(11):1362–1371. 30480030 10.1002/acn3.646 PMC6243381

[45] Beecham AH, McCauley JL. Fine-mapping array design for multi-ethnic studies of multiple sclerosis. Genes 2019 Nov 7;10(11):903. 31703377 10.3390/genes10110903 PMC6895860

